# Does the occurence of homostyly necessarily accompany the breakdown of heteromorphic incompatibility system?

**DOI:** 10.3389/fpls.2025.1402333

**Published:** 2025-02-27

**Authors:** Jing Zhao, Laiziti Kuliku, Aiqin Zhang, Fangfang Jiao, Dengfu Ren

**Affiliations:** College of Life Science and Technology, Xinjiang Key Laboratory of Biological Resources and Genetic Engineering, Xinjiang University, Urumqi, China

**Keywords:** heterostyly, variation and evolution, homostyly, heteromorphic incompatibility, reciprocal herkogamy

## Abstract

**Introduction:**

Heterostyly is a genetic polymorphism that facilitates precise pollen transfer through reciprocal herkogamy. The loss or variation of reciprocal herkogamy is usually accompanied by the breakdown of heteromorphic incompatibility system. Homostyly, which is characterized by self-compatibility and same stigma-anther height is a common floral morph in the variation and evolution of heterostyly. *Limonium aureum* is a distylous species distributed in the desert of northwest China, in which a floral morph with the same stigma-anther height (H-morph) widely distributed in the natural populations, resembling classical homostyly. The aim of this study was to clarify whether the occurrence of H-morph is also accompanied by the breakdown of heteromorphic incompatibility system, and the relationship between the H-morph and long-styled-/shortstyled-morph (L-/S-morph).

**Methods:**

The floral morphs composition and frequency, heterostylous syndrome, pollinators and visiting efficiency were investigated in five natural populations of *L. aureum* based on field observation, artificial control pollination experiment and so on.

**Results and conclusion:**

All populations were composed of L-, S- and H-morphs, except for ATS population with only H-morph, and there were significant differences in flower size parameter, fruit set, and degree of pollination limitation, while no differences among morphs within population. However, each population demonstrated dimorphic pollen-stigma morphology and a strict heteromorphic incompatibility system, especially ATS population, in which they were compatible between morphs with heteromorphic pollen-stigma morphology, regardless of the reciprocal herkogamy, and vice versa. It is speculated that the H-morph in different populations may be at different stages of heterostylous evolution. The ATS population may be a dimorphic population without reciprocal herkogamy which is in the stage before distyly formation, while the other 4 populations may be dimorphic populations with significant variation in reciprocal herkogamy which is in the stage after distyly formation. The H-morph may be caused by stigma-anther separation shortening of L- and S-morph in other 4 populations. These phenomenons that the variation of floral morph is independent of physiological incompatibility breakdown, as well as the coexistence of populations from different origins and evolutionary stages within the same species have been reported for the first time in the Plumbaginaceae.

## Introduction

It has been widely accepted that the co-evolution between angiosperms and pollinators has led to a rich diversity of species since Darwin’s time. Evolutionary ecologists have long been fascinated by the variation and evolution of floral morphology, particularly under the interaction of plants and pollinators (Zhang, 2004). Heterostyly, a genetically controlled style polymorphism, involves two (distyly) or three (tristyly) floral morphs with reciprocal arrangement of stigma and anther that promote accurate pollen transfer between morphs (Zhang et al., 2023). This adaptive strategy prevents intramorph and self-pollination, ensuring pollen grains to deposit on different parts of the pollinator’s body (Darwin, 1877; Lloyd and Webb, 1992; Barrett, 1992). The combination of stigma-anther locations and physiological incompatibility optimizes animal-mediated pollen dispersal and mating patterns. These features of heterostyly serve as classic examples of the synergistic evolution of morphology and function, highlighting the close relationship between plants and pollinators (Barrett, 2019) and also providing a paradigmatic system for studying plant-pollinator interactions. Thus, it has attracted much attention from evolutionary ecologists (Darwin, 1877; Ganders, 1979; Barrett, 1992; Lloyd and Webb, 1992; Pailler and Thompson, 1997; Costa et al., 2017b; Jia et al., 2023; Scharman and Lenhard, 2024). For example Darwin, he conducted extensive investigations in families of Primulaceae, Rubiaceae, and Lythraceae, etc, wrote the book ‘The Different Forms of Flowers on Plants of the Same Species’, and put forward the ‘hypothesis of promoting legitimate pollen (compatible pollen) transfer’ (Darwin, 1877). However, due to the widespread distribution of heterostyly, the significant differences in floral polymorphism among families and genera, and the fact that existing research has mostly focused on a few families and genera, the issues of the formation, maintenance, variation and evolution of heterostyly remain in a state of coexistence of multiple hypotheses, viewpoints and some unexplained phenomena (Charlesworth and Charlesworth, 1979; Lloyd and Webb, 1986, 1992; Brys and Jacquemyn, 2015; Simón-Porcar et al., 2015; Barrett, 2019; Zhao et al., 2023; Mora-Carrera et al., 2023; Yuan et al., 2023; Zeng et al., 2024).

Reciprocal herkogamy, a common feature of heterostyly plants, is usually accompanied by a sporophytically controlled heteromorphic incompatibility as well as a suite of ancillary morphological polymorphisms, such as stigma-pollen heteromorphism (Ganders, 1979; Lloyd and Webb, 1992; Barrett, 1992, 2019; Costa et al., 2017a; Matias et al., 2020). It plays an important role in promoting the accurate transfer of pollen (Ferrero et al., 2011; Santos-Gally et al., 2013; Zhou et al., 2015). Altering the degree of reciprocity between morphs or reducing the accuracy of reciprocal herkogamy can significantly reduce disassortative (among morphs) pollination (Jiang et al., 2018a; Brys and Jacquemyn, 2015, 2020). Due to limited mating morphs and strict selection of pollinators, heterostyly displays a high level of instability and vulnerability, characterized by morphological and physiological changes such as deviation in morph frequency, loss of reciprocal herkogamy or ancillary polymorphism, and breakdown of the heteromorphic incompatibility system (Simón-Porcar et al., 2015; Zhou et al., 2015, 2017; Costa et al., 2017b; Wu et al., 2018; Liu et al., 2022). Among them, one common type of breakdown in this system is the shift from outcrossing to strong selfing (including apomixis), involving the establishment and fixation of homostyly (distyly), semi-homostyly (tristyly), or colonization following long-distance dispersal (Guggisberg et al., 2006; Barrett, 2019). As homostyly emerges, morphological traits like flower size and reciprocal herkogamy tend to shift towards self-pollination or apomixis (Wang et al., 2021), demonstrating a strong association between morphological and physiological changes (Zhou et al., 2015; Yuan et al., 2017, 2023; Zeng et al., 2024). Particularly, Huu et al. discovered in *Primula* that the cytochrome P450 *CYP734A50* gene regulates both style length and pistil self-incompatibility in S-morph, highlighting the close connection between morphological features and physiological incompatibility at a molecular level (Huu et al., 2022). If this pattern is also present in other families and genera, it means that variations or loss of reciprocal herkogamy would be accompanied by the transfer of intramorph incompatibility or self-incompatibility. However, some existing research has drawn dubious conclusions, as the independence of morphological and physiological traits is evident in many scenarios, for instance, the two models of heterostyly formation (Baker, 1966; Charlesworth and Charlesworth, 1979; Lloyd and Webb, 1986; Pérez-Barrales et al., 2006; Ferrero et al., 2012; Santos-Gally et al., 2013). Obviously, it is worth thoroughly studying about what is the relationship between physiological and morphological characteristics, and whether the occurrence of homostyly is necessarily accompanied by the breakdown of heteromorphic incompatibility system?

Plumbaginaceae, a family with a wide distribution of distyly, comprising 27 genera and approximately 650 species (Kubitzki, 1993; Koutroumpa et al., 2018, 2021). Famous evolutionary ecologist Baker (1948, 1953a, 1966) conducted a series of studies based on wax leaf specimens, literature, and field observations. It was speculated that the most recent common ancestor of heterostyly might be self-compatible homostyly with monomorphism of pollen and stigma. Subsequently, these traits such as self-incompatibility, heteromorphic pollen and stigma, and reciprocal herkogamy emerged sequentially under selective pressure from inbreeding depression and accurate pollen transfer (Baker, 1948, 1953a, 1966). This formation pattern had laid the foundation for the proposal of “selfing avoidance hypothesis” (Charlesworth and Charlesworth, 1979). This is one of a few families that currently supports this hypothesis (Costa et al., 2019). However, the ability of this model to fully explain the development, variation, and evolution of heterostyly in Plumbaginaceae remains unknown, due to lack of evidence to underpin, especially lack of the reports on intermediate transitional populations.

*Limonium aureum* is a perennial heterostylous plant, belonging to Plumbaginaceae family. Based on a preliminary survey of five natural populations located in the southern and southwestern regions of the Tarim Basin in Xinjiang, northwest China, we found that a large number of floral morphs with the same pistil- stamen height (hereafter H-morphs) occurred in populations investigated, similar to classical self-compatible homostyly. This raises the following questions: 1) what type of style polymorphism is present in these populations; 2) what are the morphological and physiological characteristics of H-morph; 3) what kind of relationship between the H-morph and long-styled (L-) or short-styled (S-) morph; 4) whether the emergence of H-morph is necessarily accompanied by the breakdown of heteromorphic incompatibility system, etc. This plant not only provides a paradigmatic system for investigating the intermediate populations of heterostyly with H-morphs in Plumbaginaceae, but also for studying the relationship between morphological variation and physiological incompatibility in heterostyly. So, to address above questions, we carry out a series of studies on the composition and frequency of floral morph, the heteromorphism of pollen-stigma morphology, the compatibility among floral morphs, and the pollination system of representative populations across the five distinct populations of *L. aureum*. Based on these studies, the morphological and physiological characteristics of H-morph, and the relationship between morphological variation, such as the loss of reciprocal herkogamy, and physiological heteromorphic incompatibility system are explored.

## Materials and methods

### Study species and distribution areas

**Figure 1:**
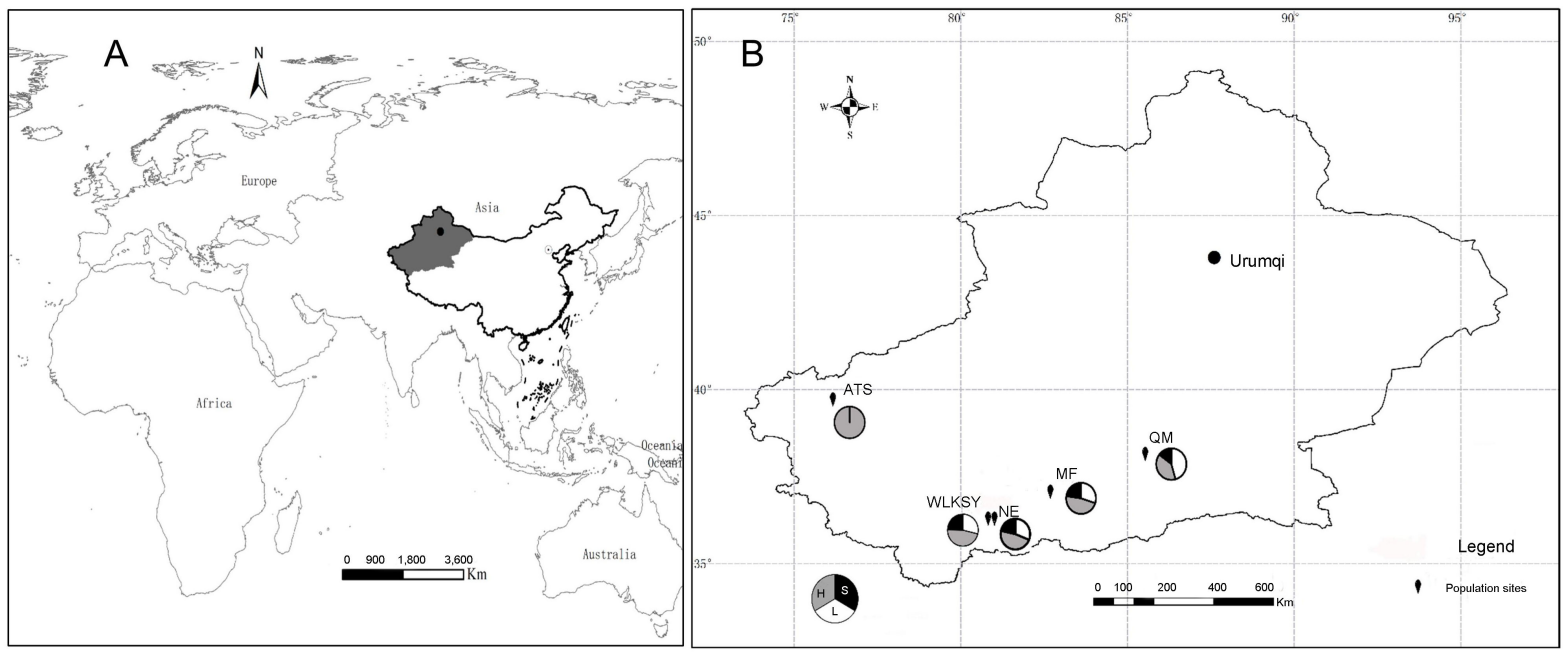
The location of five *L. aureum* populations investigated. The map was prepared using the ArcGIS ver. 10.8 (ESRI, 2021, Redland, CA, United States). **(A)** The worldwide location of sample site. **(B)** Distribution and morph composition of 5 populations in Xinjiang Province. WLKSY, Wulukesayi population; NE, Nuer population; MF, Minfeng population; QM, Qiemo population; ATS, Atushi population. For each population, the floral morph composition and frequencies are showed in the circles, L-morph (white), S-morph (black), H-morph (grey).

*Limonium aureum* (*Limonium*, Plumbaginaceae) grows in an arid desert environment with a height of 30–80cm. Each panicle-shaped inflorescence consists of 3-6 medium sized tubular flowers, with 5 filiform pistils and 5 stamens, the ovary is superior with one ovule (http://www.iplant.cn/info/). The flowering and fruiting period is from May to August. The study was conducted in Wulukesayi Township (WLKSY), Nuer Township (NE) in Cele County, Minfeng County (MF), Tula Rancho in Qiemo County (QM), and Gedaliang Township, Atushi City (ATS), Kizilsu Kirgiz Autonomous Prefecture, which located on the southern edge or southwest of the Tarim Basin (**Figure 1**, **Supplementary Table S1**). In five populations, the individuals are tall and erect, flowering in early May in ATS population, and are dwarf and creeping, flowering in June-July in the remaining four populations. These regions have a warm temperate desert climate, characterized by a single community structure and sparse vegetation.

### Composition and frequency of floral morph, as well as ancillary polymorphism

**Figure 2:**
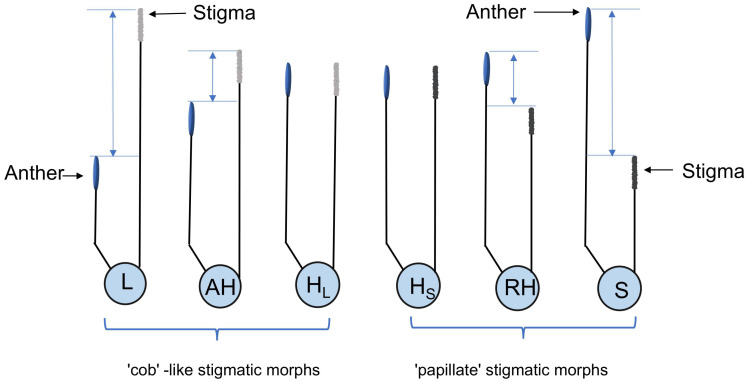
The diagram of floral morphs in *L. aureum.* L, L-morph; AH, AH-morph; H_L_, H_L_-morph; H_S_, H_S_-morph; RH, RH-morph; S, S-morph. Blue line indicate the pistil-stamen separation.

Floral morphs in five natural populations were investigated during the peak flowering period, in which 10 quadrats of 30 square meters were randomly selected, and the individuals and their floral morphs within the quadrats were counted in large populations, and all individuals and their floral morphs were counted in small populations. Due to the filamentous stigma and dimorphic stigma-pollen morphology, all morphs were categorized as following the definition of heterostyly (Barrett, 2019): (1) long-styled morph (L-morph) or short-styled morph (S-morph), where the stigma is significantly higher or lower than the anther, respectively, with an anther-stigma separation greater than 0.7 mm. (2) the floral morphs (including H_L_- and H_S_-morph) are called H-morphs, where stigma and anther are at equal height, which H_L_-morphs are consistent with L-morph, while H_S_-morphs are consistent with S-morph in stigma-pollen morphology. (3) the floral morph with approach herkogamy or reverse herkogamy is called AH- or RH-morph, where stigma is above or below anther but no contact with an anther-stigma separation less than 0.7 mm. These morphs are illustrated in **Figure 2**. Portable microscopes were used to assess stigma morphology for the statistics of H-morphs in the field.

To investigate the heteromorphism of pollen-stigma morphology, 10 individuals with L-, S-, AH-, RH-, and H-morphs (including H_L_ - and H_S_-morph) were randomly selected, and one flower was chosen from each individual. The pistils were removed and promptly fixed in 2% glutaraldehyde (0.1 mol·L^-1^ phosphate buffer), and pollen was gathered in EP tubes for air drying. Subsequently, these samples were brought to the laboratory for observation and photographed using a scanning electron microscope (LEO 1430 VP, Carl Zeiss, Oberkochen, Germany) (Costa et al., 2017a).

### Flower size parameter and the distribution of pistil-stamen height

To investigate the flower size parameter, 15-20 individuals of L-, S- and H-morphs (including H_L_-, H_S_-morphs) were randomly labeled in five populations, with 1-2 flowers were selected from each individual to measure the diameter of corolla opening, corolla tube length, corolla tube diameter, and the length of pistil and stamen using digital caliper with an accuracy of 0.02 mm (Jiao et al., 2024). The flower size parameter of the three morphs were compared within and among the five populations. Meanwhile, to investigate the distribution of pistil-stamen, and the herkogamy degree of the floral morphs, about 50 individuals with different morphs were randomly chosen from the five populations, and 1-2 flowers were randomly selected from each individual to measure the length of pistil and stamen. The degree of herkogamy is expressed by the pistil-stamen separation.

### Pollinators, visiting frequency and stigma pollen deposition

Based on previous phylogenetic analysis, the five populations are divided into two groups, in which the ATS population is an independent branch, while the other four populations with the same individual morphology and floral morph composition are clustered into one branch. Therefore, we selected one population from each branch (the ATS and WLKSY population) to represent the others. During the peak flowering period, 15-30 individuals with various morphs were randomly marked in each population, with 1-2 flowering branches were labelled for each individual. From 9:00 to 16:00, pollinator species and visiting behavior were observed for about half or an hour per individual each time. The numbers of open and visited flowers were recorded, and the visiting frequency was calculated. The cumulative observation time was no less than 15 h.

To assess pollen deposition of different floral morphs, 30 various morphic individuals were marked in 5 populations during peak flowering. Six hours post-flowering, 1-2 flowers were randomly selected from each labeled individual. Pistils were stripped to create temporary slides for counting homomorphic (incompatible) and heteromorphic (compatible) pollen grains on stigmas under a microscope (Nikon ECLIPSE E200) (Massinga et al., 2005).

### The fruit set of flower with different morph and position of inflorescence

Thirty individuals of L-, S-, and H-morphs were selected in 5 populations with 3 inflorescences being labeled in every individual. Once fruits matured, the number of fruits were counted from the first to the third or fifth flowers in each inflorescence, and then the fruit sets were calculated (Massinga et al., 2005; Jiao et al., 2024; Ren et al., 2024).

### Heteromorphic incompatibility system

During peak flowering, 30 individuals each of L-, S-, and H_S_-morph were randomly selected from the WLKSY population. Each individual was marked with 6 flowers about to open but with no dispersed pollen. The individuals were then treated as follows: (1) Intramorph pollination (emasculated, artificially pollinated with homomorphic pollen, and bagged, L×L, S×S, H_S_×H_S_); (2) Intermorph pollination (emasculated, artificially pollinated with pollen from a different floral morph, and bagged, L×S, S×L, L×H_S_, H_S_×L, H_S_×S, S×H_S_); (3) Artificial self-pollination (artificially self-pollination and bagged); (4) Apomixis (emasculated and bagged); (5) Control (open pollination without any treatment). Notably, H-morphs have two types of pollen-stigma morphology. In this study, H_S_-morph flowers were specifically chosen, and the compatible relationship of H_L_-morphs can be inferred (Massinga et al., 2005; Jiao et al., 2024; Ren et al., 2024).

Thirty individuals with H_L_- and H_S_-morphs were randomly selected from the ATS population, with 5 flowers chosen from each individual. The following treatments were conducted: 1) Intramorph pollination (emasculated, artificial pollination using homomorphic pollen and bagged, H_L_×H_L_, H_S_×H_S_); 2) Intermorph pollination (emasculated, artificial pollination using heteromorphic pollen and bagged, H_L_×H_S_, H_S_×H_L_); 3) Self-pollination (artificial self-pollination and bagged); 4) Apomixis (emasculated and bagged); 5) Control (open pollination without any treatment). Upon fruit ripening, the fruit sets of the different treatments were recorded (Rech et al., 2020; Jiao et al., 2024; Ren et al., 2024).

### Statistical analyses

The number of mating types among floral morphs (L- *vs*. S-morph, H_L_- *vs*. H_S_-morph) in different populations were compared utilizing G-test in the package “DescTools”, based on pollen-stigma morpholoy (‘cob’ *vs*. ‘papillate’) (R Core Team, 2024). The difference between flower size parameter was analyzed using generalized linear model (GLM) with normal distribution and identity link function in the “lme4” package in R (Luke, 2017). Floral morph, population, and their interaction as explanatory variables, flower size parameter as response variables. The visiting frequency was analyzed using a linear mixed-effects models (LMMs) in the package “glmmTMB”, visiting frequency as the response variable (Brooks et al., 2017), pollinator as the fixed effect, individual as the random effect, and Type II Wald chi-square tests were employed, with data transformed using artan function. The natural stigma pollen deposition was analyzed using generalized linear mixed-effects models(GLMMs) with Poisson distribution or Zero-inflated models with a log link function in the package “glmmTMB”, population, floral morph, and their interaction as explanatory variables, the number of stigma pollen grains as the response variable (Brooks et al., 2017), after checking for the absence of overdispersion in the package DHARMa (R Core Team, 2024). The null model with random effects is fitted, and the ICC value is calculated to determine whether the mixed effects model is used based on likelihood ratio tests (LRT). The fruit sets (the fruit number/flowers marked number) were analyzed using GLM with binomial distribution and logit link function in the package “glmmTMB”. Population, morph, and their interaction as explanatory variables, and fruit set as the response variable. If data showed overdispersion, choosing the Quasibinomial family for solving (Brooks et al., 2017). The fruit sets from the first to third positions of flower within inflorescence were analyzed using GLMMs with binomial distribution and logit link function in the package “glmmTMB”. The explanatory variables included population, floral position, and their interaction, fruit set as the response variable (Brooks et al., 2017). Datas from heteromorphic incompatibility system were analyzed using GLM with binomial distribution and logit link function. The explanatory variables were floral morph, treatment, and their interaction, with fruit set as the response variable in the package “lme4” (Luke, 2017). Tukey *post hoc* tests were conducted to identify significant differences between levels of explanatory variables or interactions in all models with function lsmeans in the package “lsmeans” (R Core Team, 2024). Statistical analysis was performed using R-4.3.2 (R Core Team, 2024), and software Origin 2024 was used for plotting. The values in tables and graphs are presented as mean ± SE.

## Results

### Composition and frequency of floral morph, as well as ancillary polymorphism

**Figure 3:**
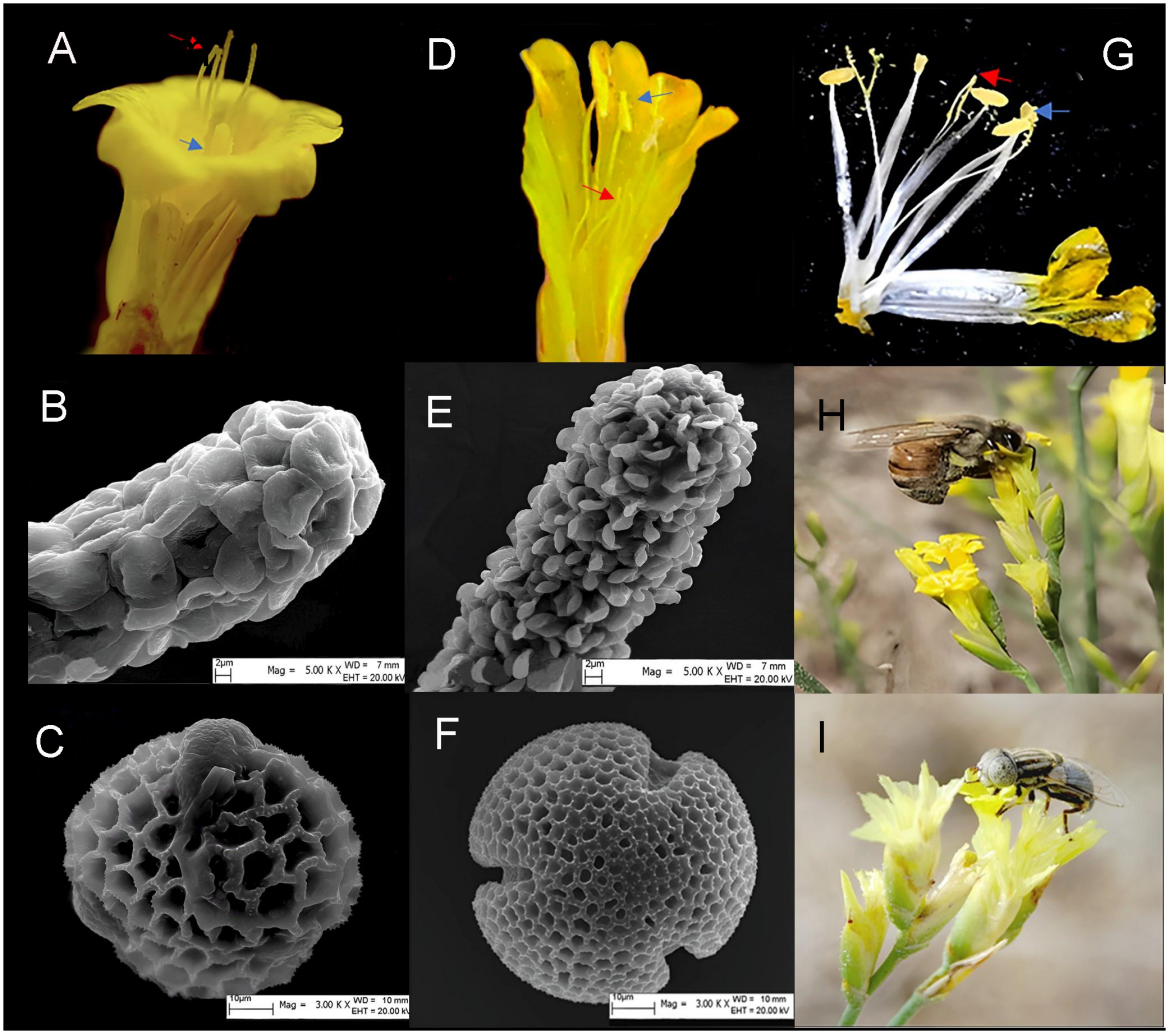
The floral morphs, stigma-pollen morphology and pollinators of *L. aureum*. **(A)** L-morph. **(B)** The morphology of stigma in L-, AH-, H_L_-morph flowers. **(C)** The morphology of pollen in L-, AH-, H_L_-morph flowers. **(D)** S-morph. **(E)** The morphology of stigma in S-, RH-, H_S_- morph flowers. **(F)** The morphology of pollen in S-, RH-, H_S_- morph flowers. **(G)** H-morph. Blue arrow indicate anther, red arrow indicate stigma. **(H)** *Apis mellifera*. **(I)** Fly.

The five populations showed two types of floral morph compositions. The ATS population is dominated by H-morphs (including H_L_- and H_S_-morphs), with a few RH-, and AH-morphs, while the other populations consisted of L-, S-, H-, AH-, and RH-morphs (**Figures 2**, **3A, D, G**). Scanning electron microscopy (SEM) imaging revealed that five populations all exhibited dimorphism of pollen-stigma morphology. The stigma epidermal cells of L-, AH-, and H_L_-morphs appeared ‘cob’ -like, with coarse reticulate exine ornamentation on pollen grains (referred to as ‘cob’ stigma) (**Figures 3B, C**). Conversely, the stigma epidermal cells of S-, RH-, and H_S_-morphs were papillate, with finely reticulate exine ornamentation on pollen grains (referred to as ‘papillate’ stigma) (**Figures 3E, F**).

**Figure 4:**
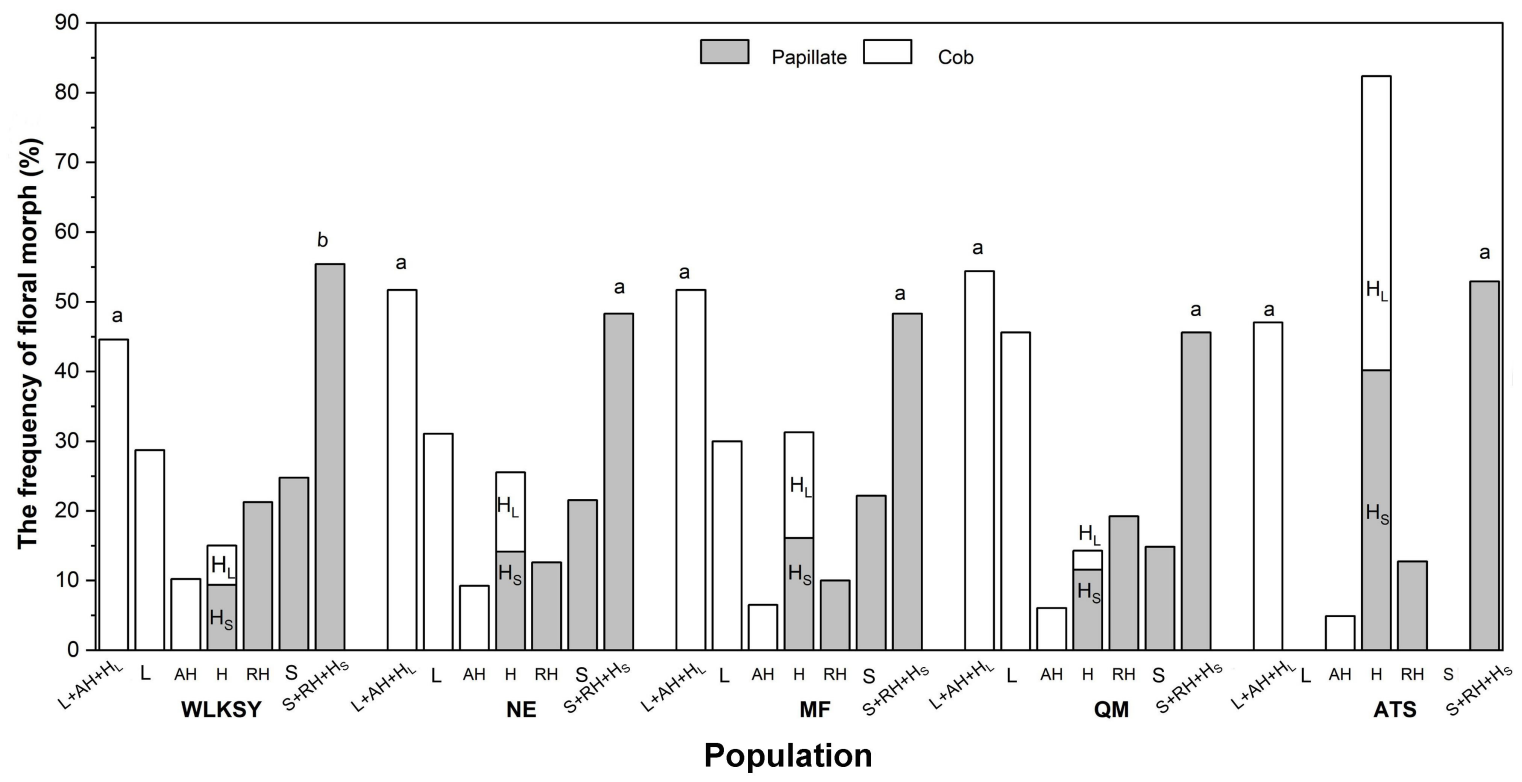
The floral morphs composition and frequency of *L. aureum* in different populations. Different letters indicate significant difference (*P*<0.01), the same letters indicate no significant difference within population (*P>*0.05).

In the ATS population, H-morph displayed two distinct types of pollen and stigma, with a 1:1 quantity ratio (G=3.353, *P*=0.552). Among the other four populations, L-morphs were dominant in ‘cob’ stigmatic morphs (the ratio of L-morph, WLKSY: 28.75%; NE: 31.08%; MF: 30%; QM: 45.60%, **Figure 4**), while the frequencies of S-, RH-, and H_S_-morphs were similar in ‘papillate’ stigmatic morphs (the ratio of S-morph, WLKSY: 24.79%; NE: 21.54%; MF: 22.17%; QM: 14.84%), indicating a difference in variation and differentiation degree between L- and S-morphs (**Supplementary Table S1**; **Figure 4**). However, the floral morphic frequency with ‘papillate’ and ‘cob’ stigma was 1:1 in all populations (NE: G=0.372, *P*=0.542; MF: G=0.278, *P*=0.598; QM: G=1.408, *P*=0.235), except WLKSY (G=5.644, *P*=0.018) (**Supplementary Table S1**; **Figure 4**).

### Flower size parameter and the distribution of pistil-stamen height

Among L-, S-, and H-morphs within populations of WLKSY, NE, MF, and QM, there were no significant differences in corolla opening diameter (WLKSY: Waldχ^2^ = 0.824, *P*=0.662; NE: Waldχ^2^ = 1.012, *P*=0.603; MF: Waldχ^2^ = 0.565, *P*=0.754; QM: Waldχ^2^ = 0.880, *P*=0.644), corolla tube length (WLKSY: Waldχ^2^ = 0.745, *P*=0.689; NE: Waldχ^2^ = 0.437, *P*=0.804; MF: Waldχ^2^ = 0.393, *P*=0.822; QM: Waldχ^2^ = 1.220, *P*=0.543), and corolla tube diameter (WLKSY: Waldχ^2^ = 2. 282, *P*=0.319; NE: Waldχ^2^ = 0.986, *P*=0.611; MF: Waldχ^2^ = 2.777, *P*=0.249; QM: Waldχ^2^ = 0.956, *P*=0.620). However, there were significant differences in the heights of pistil (WLKSY: Waldχ2 = 77.895, *P*<0.001; NE: Waldχ2 = 143.766, *P*<0.001; MF: Waldχ2 = 63.523, *P*<0.001; QM: Waldχ2 = 88.874, *P*<0.001) and stamen (WLKSY: Waldχ2 = 90.715, *P*<0.001; NE: Waldχ2 = 106.598, *P*<0.001; MF: Waldχ2=55.690, *P*<0.001; QM: Waldχ2 = 105.453, *P*<0.001) (**Table 1**). The heights of pistil and stamen in H-morph fell between those of L- and S-morphs in four populations of WLKSY, NE, MF, QM, and there was reciprocal herkogamy between L- and S-morphs.

**Table 1:** The flower size parameter in different populations of *L. aureum*.

Flower size parameter	Morph	WLKSY	NE	MF	QM	ATS
(mm)		Mean ± SE	Mean ± SE	Mean ± SE	Mean ± SE	Mean ± SE
Corolla opening diameter	L	4.67 ± 0.06^a^	4.36 ± 0.10^b^	4.16 ± 0.08^b^	4.81 ± 0.09^a^	
	S	4.61 ± 0.07^b^	4.32 ± 0.07^c^	4.24 ± 0.06^c^	4.91 ± 0.04^a^	
	H_L_+Hs	4.56 ± 0.08^c^	4.43 ± 0.08^c^	4.19 ± 0.06^d^	4.88 ± 0.09^b^	5.13 ± 0.11^a^
Corolla tube length	L	6.84 ± 0.07^a^	6.72 ± 0.11^a^	6.22 ± 0.08^b^	6.90 ± 0.12^a^	
	S	6.76 ± 0.11^ab^	6.63 ± 0.13^b^	6.30 ± 0.12^c^	6.99 ± 0.09^a^	
	H_L_+Hs	6.70 ± 0.10^c^	6.73 ± 0.12^c^	6.21 ± 0.11^d^	6.82 ± 0.13^b^	11.07 ± 0.15^a^
Corolla tube diameter	L	0.99 ± 0.03^c^	1.31 ± 0.03^a^	1.18 ± 0.04^b^	1.15 ± 0.05^b^	
	S	1.07 ± 0.05^bc^	1.34 ± 0.04^a^	1.19 ± 0.03^b^	1.10 ± 0.04^b^	
	H_L_+Hs	1.04 ± 0.02^c^	1.35 ± 0.03^a^	1.25 ± 0.05^b^	1.14 ± 0.02^c^	1.07 ± 0.03^cd^
Pistil height	L	**7.21 ± 0.09** ^b^	**7.70 ± 0.12** ^a^	**7.15 ± 0.07** ^b^	**7.66 ± 0.11** ^a^	
	S	**5.98 ± 0.11** ^b^	**6.04 ± 0.10** ^b^	**6.05 ± 0.12** ^b^	**6.37 ± 0.08** ^a^	
	H_L_+Hs	**6.63 ± 0.08** ^d^	**6.99 ± 0.07** ^c^	**6.55 ± 0.11** ^d^	**7.26 ± 0.10** ^b^	10.45 ± 0.10^a^
Stamen height	L	**5.94 ± 0.76** ^b^	**6.18 ± 0.10** ^ab^	**5.99 ± 0.09** ^ab^	**6.20 ± 0.11** ^a^	
	S	**7.17 ± 0.09** ^b^	**7.49 ± 0.10** ^a^	**6.92 ± 0.11^b^**	**7.47 ± 0.10^a^**	
	H_L_+Hs	**6.75 ± 0.06** ^c^	**7.13 ± 0.08** ^b^	**6.72 ± 0.11^c^**	**7.22 ± 0.10^b^**	10.20 ± 0.12^a^
Pistil-Stamen separation	L	1.27 ± 0.10^ab^	1.52 ± 0.12^a^	**1.17 ± 0.07** ^b^	**1.46 ± 0.14** ^a^	
	S	1.18 ± 0.11^b^	1.45 ± 0.14^a^	**0.87 ± 0.09** ^c^	**1.10 ± 0.07** ^bc^	

In the same column of the same flower size parameter, bold values indicate the significant difference among floral morphs within populations (*P*<0.05); in the same row, the same letter indicates that there is no significant difference within floral morphs of the same flower size parameter among populations (*P*>0.05).

Among five populations, the flower size in ATS population was significantly larger than the corresponding parameter of other populations in corolla opening diameter (H-morph: Waldχ2 = 92.611, *P*<0.001), corolla tube length (H-morph: Waldχ2 = 1295.724, *P*<0.001), pistil height (H-morph: Waldχ2 = 1097.512, *P*<0.001), and stamen height (H-morph: Waldχ2 = 1002.481, *P*<0.001) (**Tables 1**, **2**).

**Table 2:** The comparison of flower size parameter among morphs, populations and their interactions using generalized linear model (GLM).

Dependent variable	Source	df	Wald χ^2^	*P*
Corolla opening diameter	Morph	2	0.226	0.893
	Population	4	179.128	**< 0.001**
	Morph×Population	6	3.054	0.802
Corolla tube length	Morph	2	0.681	0.711
	Population	4	1337.021	**< 0.001**
	Morph×Population	6	2.114	0.909
Corolla tube diameter	Morph	2	2.467	0.291
	Population	4	123.75	**< 0.001**
	Morph×Population	6	4.534	0.605
Pistil height	Morph	2	361.835	**< 0.001**
	Population	4	1120.639	**< 0.001**
	Morph×Population	6	12.223	0.057
Stamen height	Morph	2	351.672	**< 0.001**
	Population	4	1027.799	**< 0.001**
	Morph×Population	6	6.784	0.341
Pistil/Stamen separation	Morph	1	7.469	**0.006**
	Population	3	20.417	**< 0.001**
	Morph×Population	3	2.956	0.398

The significance of bold values is that the variation between different treatments has significant difference.

**Figure 5:**
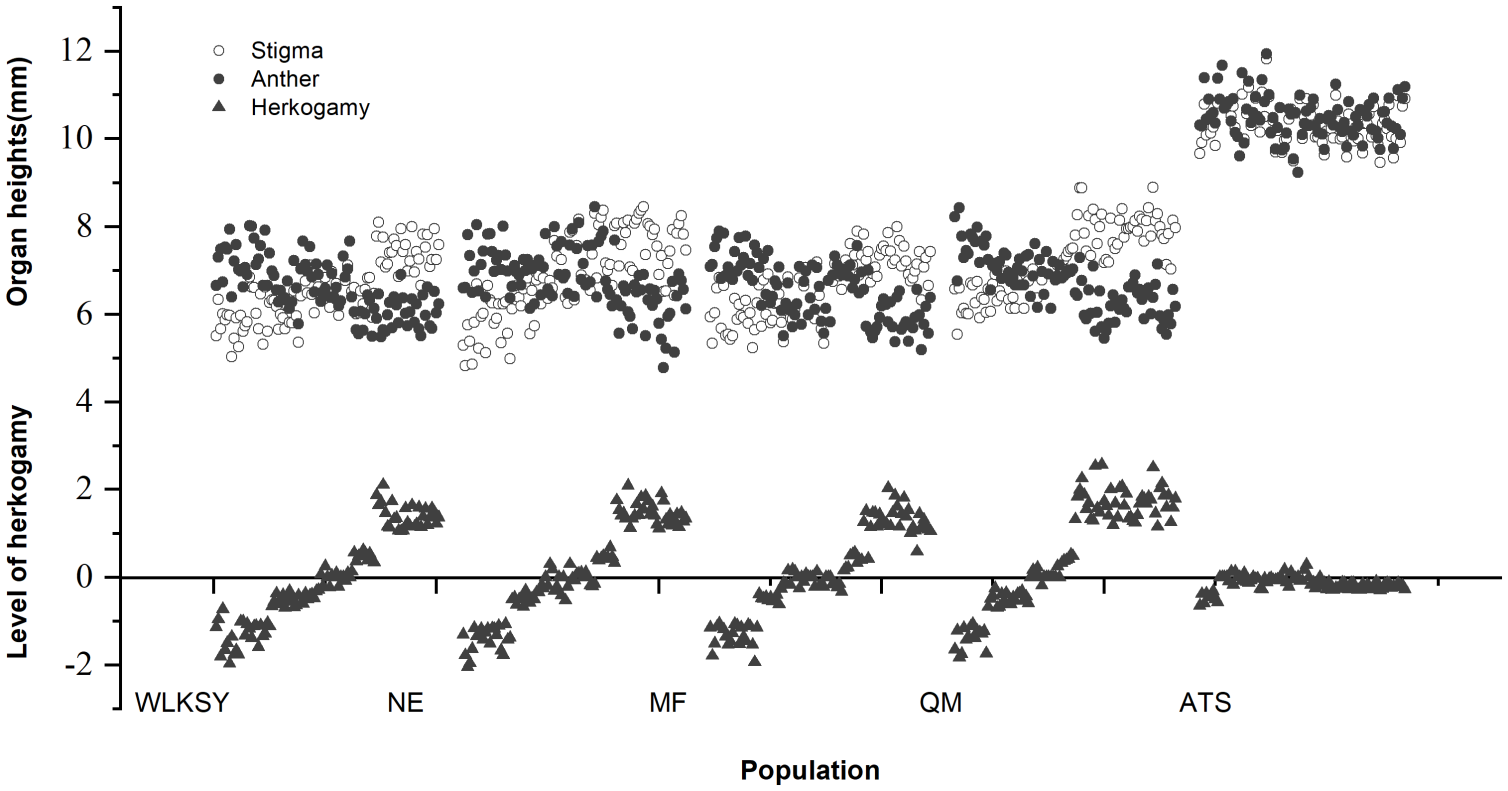
The distribution of pistil and stamen and level of herkogamy in different populations of *L. aureum*.

The distribution of pistil and stamen and level of herkogamy of five populations were expressed based on the pistil-stamen height of different floral morphs (**Figure 5**). The stigma and anther heights in the WLKSY, NE, MF, and QM populations were similar and notably lower than those in the ATS population (The stigma and anther heights in ATS: >10mm, others: 6-8mm).

In the five populations, corolla opening diameter, diameter and length of the corolla tube were not influenced by morph and morph×population, but rather by population alone. Both pistil and stamen heights, as well as pistil-stamen separation were influenced by morph and population, but not by their interaction (morph×population) (**Table 2**). Sex organ height was not affected by sex organ type or sex organ type×population, but rather by sex organ type×morph and sex organ type×morph×population interactions (**Table 3**).

**Table 3:** The comparison of sex organ height among four populations using generalized linear model (GLM).

Dependent variable	df	Wald χ^2^	*P*
Population	3	105.850	**< 0.001**
Morph	2	22.670	**< 0.001**
Sex organ type	1	0.832	0.362
Morph× Sex organ type	2	712.257	**< 0.001**
Morph×Population	6	6.618	0.358
Population×Sex organ type	3	2.655	0.448
Morph×Population×Sex organ type	6	13.282	**0.039**

The significance of bold values is that the variation between different treatments has significant difference.

### Pollinators, visiting frequency and stigma pollen deposition

**Figure 6:**
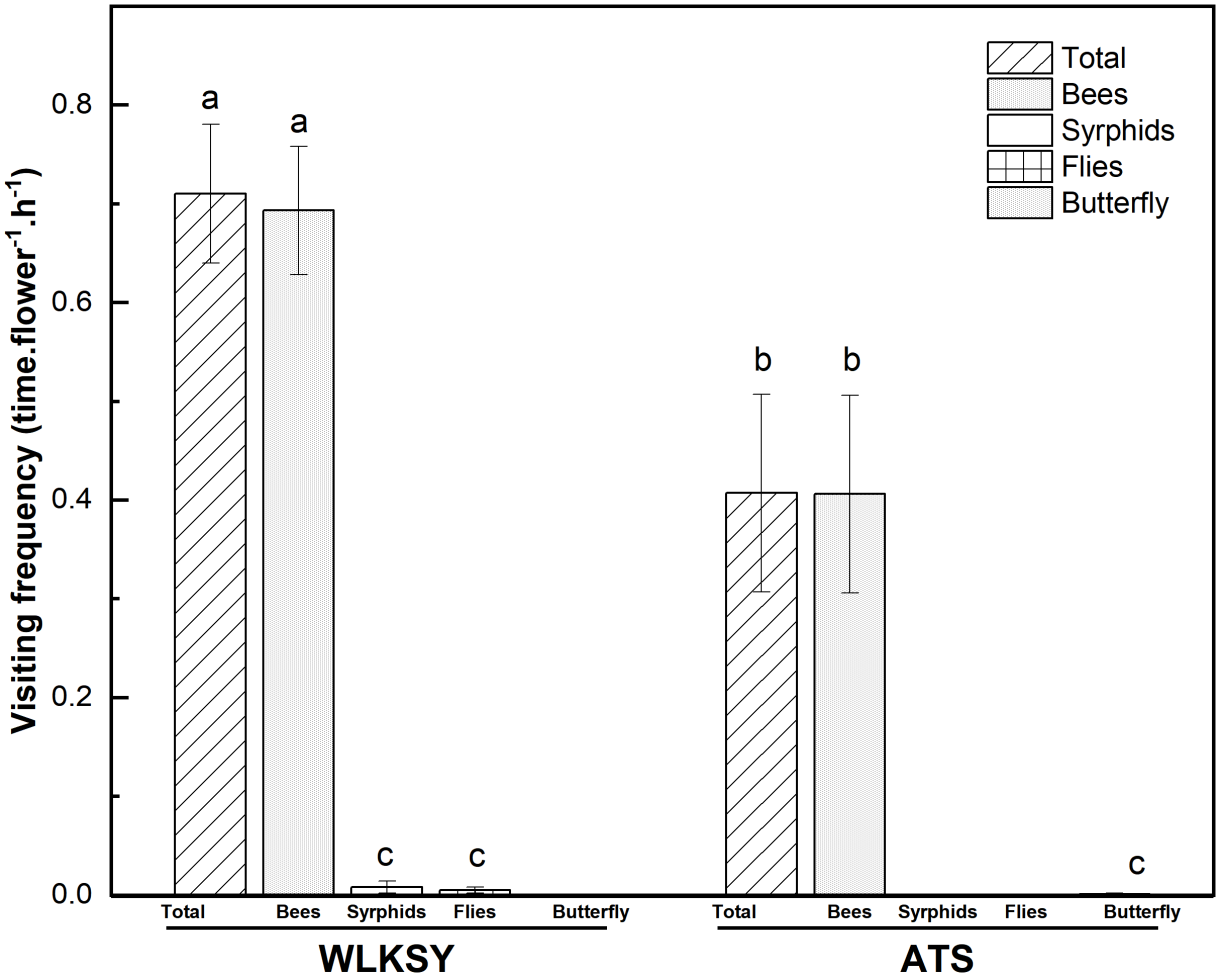
The visiting frequency in two populations of *L. aureum* (Bars indicate mean ± SE). Different letters indicate significant difference (*P*<0.001), the same letters indicate no significant difference within population (*P>*0.05).

In ATS population, the main pollinator is bees with a total visiting frequency of 0.407 ± 0.10 times·flower^-1^·h^-1^, in which bees and butterflies were 0.406 ± 0.1 and 0.001 ± 0.001 times·flower^-1^·h^-1^, respectively, based on 19.5 hours of observation (Waldχ^2^ = 17.038, *P*<0.001, **Figure 6**).

By an observation of 22.25 hours in WLKSY population, the total visiting frequency was 0.71 ± 0.07 times·flower^-1^·h^-1^, in which bees, Syrphids, and flies were 0.693 ± 0.065 times·flower^-1^·h^-1^, 0.008 ± 0.006 times·flower^-1^·h^-1^, and 0.005 ± 0.003 times·flower^-1^·h^-1^, respectively (Waldχ^2^ = 227.019, *P*<0.001, **Figures 3H, I**, **6**). Visiting frequency was significantly higher in WLKSY compared to ATS (Waldχ^2^ = 7.552, *P*<0.01).

The total stigma pollen deposition of 5 populations were significantly different (Waldχ^2^ = 1909.85, *P*<0.001), in which the quantity of compatible pollen grains in WLKSY was notably higher compared to the other four populations (Waldχ2 = 1541.1, *P*<0.001), followed by ATS and then the remaining 3 populations of NE, MF and QM (**Table 4**). In WLKSY, the compatible pollen counts for L-, H-, and S-morphs were 11.34 ± 1.59, 11.63 ± 2.11, and 7.80 ± 0.74, respectively (Waldχ2 = 30.656, *P*<0.05). A significant difference was observed between L- and S-morphs (*P*<0.05). The compatible pollen grains in ATS were 1.48 ± 0.17, significantly lower than that in WLKSY, but notably higher than that in the 3 populations of NE, MF and QM (All *P*<0.001). The compatible pollen counts in of NE, MF and QM populations were relatively low, in which significant differences were observed in MF and QM (All *P*<0.01), but not in NE (Waldχ2 = 0.492, *P*=0.782). In MF, L- and S-morphs showed significant differences (*P*<0.01), while in QM, L- and H-morphs as well as S- and H-morphs exhibited significant differences (All *P*<0.05) (**Table 4**). The quantity of compatible pollen grains was significantly influenced by population (Waldχ2 = 1038.194, *P*<0.001) and morph (Waldχ2 = 19.767, *P*<0.001), and not affected the interaction between population and morph (Waldχ2 = 26.340, *P*=0.673).

**Table 4:** The analysis of natural stigma pollen number from 5 populations of *L. aureum*.

Population	Morph	Compatible pollen	Total pollen
WLKSY	L	11.34 ± 1.59^a^	97.29 ± 3.30^a^
	H	11.63 ± 2.11^a^	73.26 ± 7.71^b^
	S	7.80 ± 0.74^b^	29.94 ± 1.24^c^
NE	L	0.10 ± 0.06^a^	90.80 ± 4.84^a^
	H	0.13 ± 0.06^a^	61.40 ± 10.91^b^
	S	0.17 ± 0.07^a^	19.13 ± 1.05^c^
MF	L	0.30 ± 0.13^b^	47.98 ± 4.51^a^
	H	0.8 ± 0.21^a^	25.35 ± 4.01^b^
	S	1.03 ± 0.23^a^	9.23 ± 0.87^c^
QM	L	0.56 ± 0.13^a^	78.06 ± 5.36^a^
	H	0.41 ± 0.11^b^	35.53 ± 4.27^b^
	S	0.44 ± 0.12^b^	34.00 ± 1.90^c^
ATS	L	0	0
	H	1.48 ± 0.17	78.31 ± 5.66
	S	0	0

In the same column, the same letter indicates that there is no significant difference between floral morphs within population (*P*>0.05).

### The fruit set of flower with different morph and position of inflorescence

**Figure 7:**
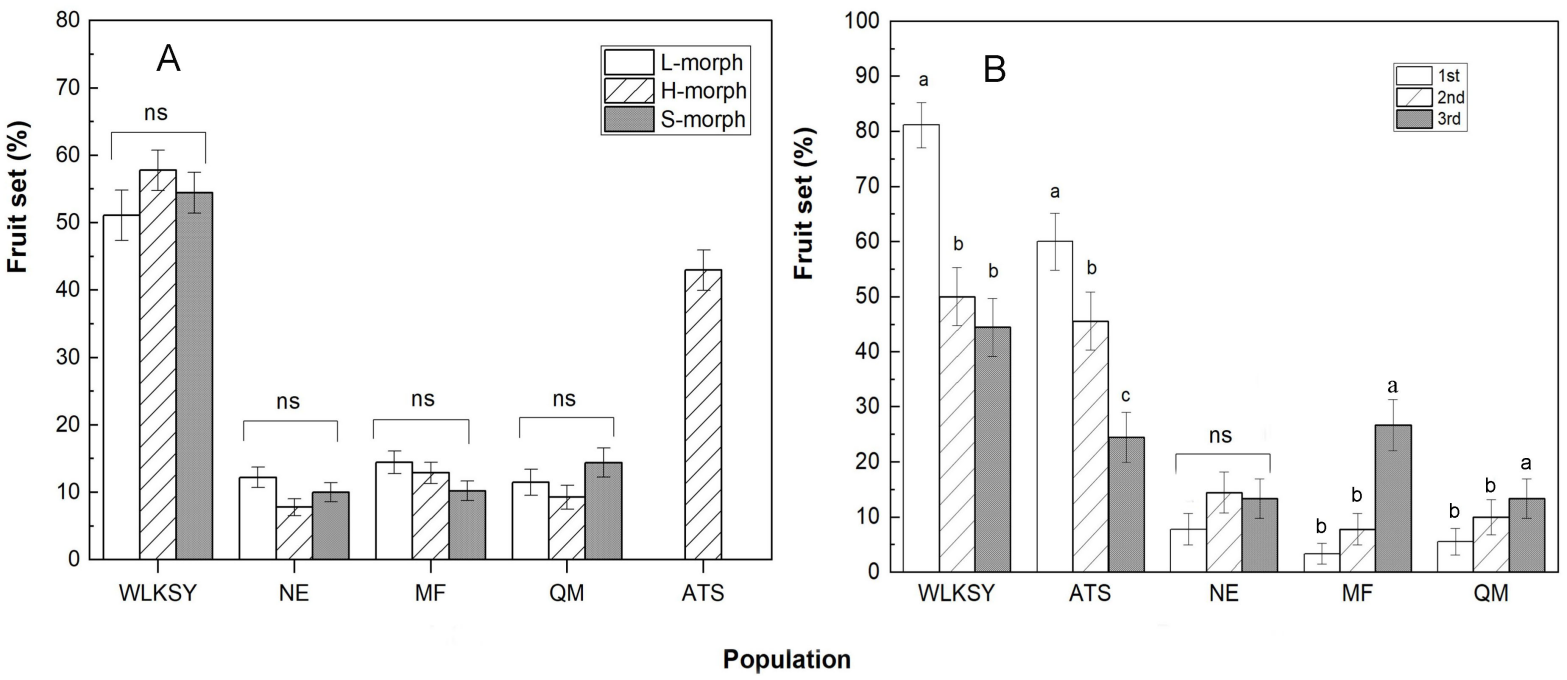
The Fruit set in different floral morph and position in inflorescence within population of *L. aureum.* **(A)** The fruit set of three morphs. **(B)** The fruit set of 1st-3rd position flowers in inflorescence. “ns” indicates the fruit set of different morph has no significant difference within population, *P*>0.05. The same letters represent no significant difference (*P>*0.05), while different letters represent significant difference in the same population (*P<*0.001), Bars indicate mean ± SE. The fruit set of 1st-3rd were compared among populations based on H-morphs.

Through the comparison of fruit set, there were no significant differences between morphs within each population (All *P*>0.05, **Figure 7A**), but it was notably higher in WLKSY (fruit set: 51.11 ± 3.73% -57.78 ± 3.00%) than that in the other 4 populations (Waldχ2 = 624.91, *P*<0.001), followed by ATS (fruit set: 42.96 ± 3.01%) and then the remaining 3 populations (fruit set: at around 10%). The fruit set was significantly impacted by the population (Waldχ2 = 598.78, *P*<0.001), but not by morph (Waldχ2 = 0.63, *P*=0.73), and the interaction between population and morph (Waldχ2 = 12.44, *P*=0.053).

Within the inflorescence, the fruit sets of the first, second, and third flower (from outside to inside) were 82.22 ± 4.05%, 47.78 ± 5.30%, 45.56 ± 5.28%, respectively in the WLKSY population (Waldχ2 = 28.61, *P*<0.001), and were 60.00 ± 5.19%, 45.56 ± 5.28%, 23.33 ± 4.48%, respectively in the ATS population (Waldχ2 = 23.602, *P*<0.001). Both populations had the highest fruit set in the first position, followed by the second and third (**Figure 7B**). The remaining three populations had the lowest fruit set. The fruit sets of different position were significantly affected by the population (Waldχ2 = 171.918, *P*<0.001), the floral position (Waldχ2 = 10.742, *P*<0.01), and their interaction (population×floral position) (Waldχ2 = 66.343, *P*<0.001).

### Heteromorphic incompatibility system

**Figure 8:**
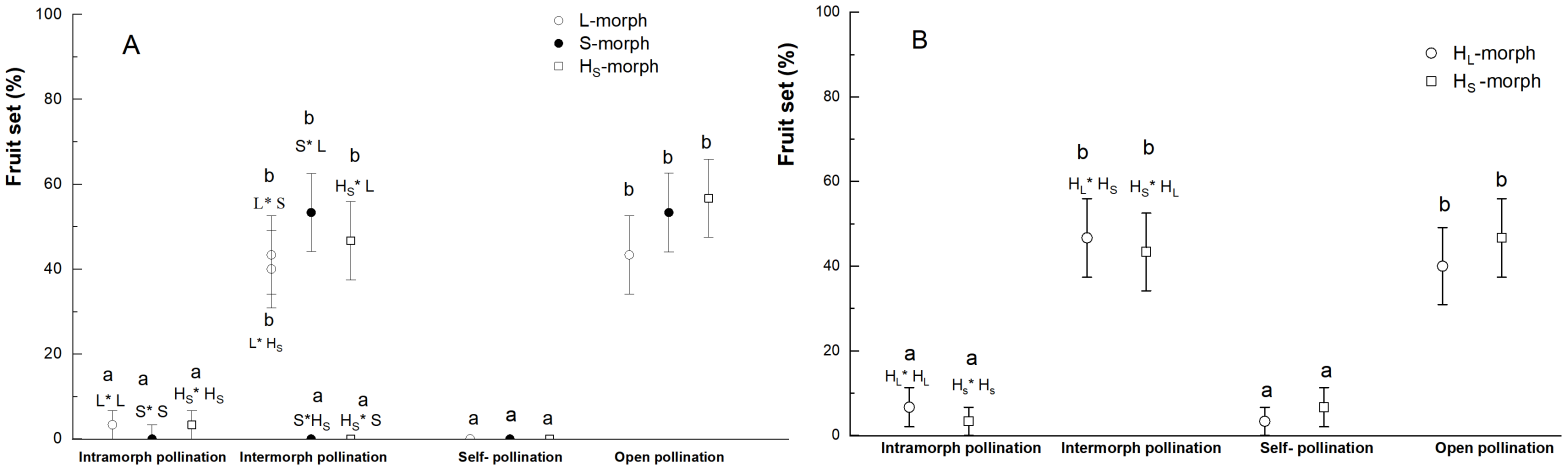
The fruit set of two populations under different pollination treatments in *L. aureum.* **(A)** WLKSY and **(B)** ATS. The same letters represent no significant difference between mating type or treatment (*P>*0.05), while different letters represent significant difference (*P<*0.001), Bars indicate mean ± SE.

The *L. aureum* species exhibited no apomixis, with self and intramorphic incompatibility, so the flowers produced no or a few fruits, after emasculation and bagging, as well as intramorphic and artificial self-pollination (**Figures 8A, B**). For example, the fruit sets of artificial self-pollination were 0, of intramorph pollination (S×S, L×L, H_S_×H_S_) were 0, 3.33 ± 3.33%, and 3.33 ± 3.33%, respectively (Waldχ2 = 1.644, *P*=0.439) in the WLKSY population, and the fruit sets of intramorph pollination (H_L_×H_L_, H_S_×H_S_) were 6.67 ± 4.63% and 3.33 ± 3.33% (Waldχ2 = 0.353, *P*=0.552), of artificial self-pollination of H_L_- and H_S_- morph were 3.33 ± 3.33% and 6.67 ± 4.63%, respectively (Waldχ2 = 0.353, *P*=0.552) in the ATS population. Intermorph pollination, such as L×S (43.33 ± 9.20%), S×L (56.67± 9.20%), L×H_S_ (40.0 ± 9.10%), H_S_×L (43.33 ± 9.20%), there were no significant difference in fruit set (*P*>0.05), and they were higher than that of S×H_S_ (0) and H_S_×S (0) (Waldχ2 = 58.037, *P*<0.001) in WLKSY population.

In the ATS population, the fruit sets of H_L_×H_S_ and H_S_×H_L_ were 46.67 ± 9.26% and 43.33 ± 9.20%, respectively (Waldχ2 = 0.067, *P*=0.795), with no significant difference compared with natural contrast (H_L_: 40.00 ± 9.10%; H_S_: 46.67 ± 9.26%; Waldχ2 = 0.272, *P*=0.602), There was no significant difference among the four different pollination treatments (Waldχ2 = 0.430, *P*=0.934, **Figure 8B**).

The fruit sets were significantly impacted by the treatments (WLKSY: Waldχ2 = 161.299, *P*<0.001; ATS: Waldχ2 = 85.127, *P*<0.001), unaffected by morphs (WLKSY: Waldχ2 = 1.737, *P*=0.420; ATS: Waldχ2 = 0.028, *P*=0.866), and their interaction between morph and treatment (WLKSY: Waldχ2 = 6.895, *P*=0.548; ATS: Waldχ2 = 1.025, *P*=0.906). In conclusion, *L. aureum* exhibited self-incompatibility and a strict heteromorphic incompatibility system, without apomixis, in which the morphs with heteromorphic stigma-pollen morphology were compatible, and vice versa.

## Discussion

Based on the study of heterostyly syndrome and pollination system in *L. aureum*, an unusual floral morph and some heterostylous populations at different stages of evolution were revealed within the Plumbaginaceae. In the five populations investigated, only the ATS population mainly consisted of H-morph with the same stigma-anther height, while the remaining four populations were mainly composed of L-morph, S-morph, H-morph across, but the pollen-stigma morphology of all were dimorphic. Those morphs with heteromorphic stigma-pollen morphology were compatible, and vice versa. Reciprocal herkogamy between morphs was no longer a necessary condition for compatibility, and intramorph and selfing pollinations were incompatible in this species. Unlike classic homostyly, the occurrence of H-morph was not accompanied by the breakdown of heteromorphic incompatibility system (Zhou et al., 2015; Yuan et al., 2017, 2023; Zhang et al., 2021; Zeng et al., 2024).

### Floral polymorphism and its relationship

The heterostyly syndrome is composed of a suite of features such as reciprocal herkogamy, ancillary polymorphism of pollen-stigma morphology, and the heteromorphic incompatibility system (Ganders, 1979; Dulberger, 1992; Barrett, 2019). We have down research on related traits in *L. aureum*. From the results of flower size parameter and ancillary polymorphism, there were only two types of pollen-stigma morphology in all populations, in which H-morph was either consistent with L- or S-morph (**Figure 3**), and without significant difference in flower size parameter within population (**Table 1**). Moreover, the compatibility relationship between floral morphs was closely related to pollen-stigma morphology, but not the reciprocal herkogamy (**Figure 5**). So H-morph differ from the classic homostyly, which lacks self-compatibility and floral features promoting self-pollination (**Table 1**; **Figure 8**). Although H-morphs were widely distributed in five populations, the heteromorphic incompatibility system still be maintained, especially in ATS population with mainly H-morph (**Figure 8B**). This phenomenon that the variation or loss of reciprocal herkogamy was not accompanied by the breakdown of physiological heterostylous incompatibility was also found in other species of the *Limonium* (Ayiguli et al., 2021; Ren et al., 2024; Jiao et al., 2024).

Floral polymorphisms, function, and the relationship between morphology and physiology has been one of the focus issues of studies in heterostyly (Sicard and Lenhard, 2011; Zhao et al., 2023). Based on the “hypothesis of promoting compatible pollen transfer” (Darwin, 1877), it can be deduced that the morphological reciprocal herkogamy and the physical intramorph and self-incompatibility are two distinct mechanisms promoting outcrossing (Baker, 1966; Charlesworth and Charlesworth, 1979; Ganders, 1979; Barrett, 1992). For example, among different evolutionary models of explaining heterostyly formation, the “selfing avoidance hypothesis” (Charlesworth and Charlesworth, 1979) and “pollen transfer hypothesis” (Lloyd and Webb, 1992), a common point of view were the build-up of distyly in several steps, in which the reciprocal herkogamy and physiological heterostylous incompatibility appeared in different stages. These hypothesis fully reflects the independence of morphological and physiological traits. However, different views was hold that the morphological and physiological characteristics of heterostyly were closely related, and the real significance of morphological differences between stamen and pistil lied in the resulting physiological differences, and that there must be a developmental link between the two characteristics (Mather and de Winton, 1941). Notable is, the latter is widely supported and verified in subsequent research (Zhou et al., 2015; Jiang et al., 2018b; Wu et al., 2018; Huu et al., 2022; Mora-Carrera et al., 2023, 2024; Zeng et al., 2024), in particular, the molecular evidence from *Primula* provides strong support (Huu et al., 2022). However, direct evidence for the former is lacking, although there is some support in the studies of *Narcissus*, *Lithodora* and *Glandora* (Pérez-Barrales et al., 2006; Ferrero et al., 2012; Santos-Gally et al., 2013). But it is mostly based on the of existing hypotheses (Darwin, 1877; Charlesworth and Charlesworth, 1979; Lloyd and Webb, 1992) or the result analysis of phylogenetic relationship (Pérez-Barrales et al., 2006; Ferrero et al., 2012; Santos-Gally et al., 2013). For instance, Ferrero et al. (2012) proposed the evolution towards reciprocal herkogamy was not associated with the acquisition of incompatibility by reconstructing the phylogenetic trees of two genera of *Lithodora* and *Glandora* (Ferrero et al., 2012). Our study result on *L. aureum* provides a strong evidence for the former view, that is, the occurrence of H-morph is not accompanied by the breakdown of heteromorphic incompatibility system.

Compared with reciprocal herkogamy, the function of ancillary polymorphism is less concerned, although “the morphological complementarity hypothesis” was proposed for many years (Dulberger, 1975, 1992). In *L. aureum*, the dimorphism of pollen-stigma morphology was very typical and showed a significant correlation with the compatibility between floral morphs (**Figure 8**). Although the function of promoting pollen transfer has been verified in dimorphic *Armeria maritima* and *A. pubigera* (lacking reciprocal herkogamy), and distylous *Limonium vulgare* (Plumbaginaceae) (Costa et al., 2017a), whether the variation or loss of the dimorphism of pollen-stigma morphology is accompanied by the breakdown of physiological heterostylous incompatibility is still unclear.

The *Limonium* is one of the most widely distributed and diverse genera in the Plumbaginaceae (Koutroumpa et al., 2018). The Mediterranean is a center of diversity for the genus (Baker, 1948, 1953b, 1966; Koutroumpa et al., 2021), in which most of species are present in the form of homostyly with dimorphic pollen-stigma morphology and self-incompatibility (Costa et al., 2019). Among the five populations investigated, the floral composition and syndrome of the ATS population were consistent with that of the species in the Mediterranean, and distinct from the other four populations. In order to explore the factors affecting floral variation, we investigated pollination systems and fruit sets of five populations. The result showed that most populations have varying degrees of pollination restriction, and lacking of long-tongued insects. For example, the major pollinator of the WLKSY and ATS populations are both *Apis mellifera* L. From the pollination efficiency, S-morphs have a lower compatible pollen load compared with L- and H-morphs in WLKSY population. In ATS population, the pollination efficiency and fruit sets were lower than that of WLKSY population. Moreover, we noticed that the corolla tubes of flower visited by bees were split, and *Apis mellifera* tended to visit other species blooming at the same time. It is speculated that the longer corolla tubes limited the species and pollination efficiency of pollinators (Santos-Gally et al., 2013). Because of the longer corolla tube, long-tongued (LT) insects are frequently ideal pollinators, whereas short-tongued (ST) insects are usually ineffective or inefficient due to low contact probability with low sex organs (Arroyo and Dafni, 1995; Arroyo et al., 2002; Jiao et al., 2024). From the floral morph frequency, the degree of variation in S-morphs were greater than that of L-morphs in all populations except for ATS population. It reflected that S-morphs were subjected to greater selection pressure. In terms of promoting the transfer of compatible pollen. H-morphs can not only avoid the disadvantage of retraction of lower sex organs in S-morphs, but also promote pollen distribution in L-morphs (Jiao et al., 2024; Ren et al., 2024). Based on the morphologies of pollen and stigma, and the compatibility between the morphs, it can be inferred that the H-morph flowers are derived from the shortening of between the pistil-stamen separation of L- and S-morphs in four populations of WLKSY, MF, QM, and NE, due to lack of pollinators or being affected the selective pressure to promote the efficient transfer of compatible pollen. These four populations and the ATS population may be in different stages of heterostyly evolution. These findings support the selfing-avoidance hypothesis (Charlesworth and Charlesworth, 1979) and the formation and evolutionary sequence model of heterostyly proposed by Barrett (Barrett, 2019). This co-existence of populations with different evolutionary sequences has been reported for the first time in Plumbaginaceae.

This paper reveals an important phenomenon that the occurrence of H-morph or the loss of reciprocal herkogamy is not accompanied by the breakdown of heteromorphic incompatibility system through a series of studies. At the same time, it also provides some evidences for in-depth understanding of the “selfing avoidance hypothesis” and the relationship between morphology and physiology in heterostyly. However, some questions remain to be investigated later, such as what are the factors affecting the formation of H-morph, whether H-morph has the same origin in different populations, such as ATS and the other four populations, and what is the evolutionary relationship among the different populations and so on.

## Conclusions

The study of five populations of *Limonium aureum* has shown that morphological variation may be at various stages of heterostyly evolution, with each population displaying dimorphism in pollen and stigma morphology, and a strict heteromorphic self-incompatibility system. The morphological variation (the loss of reciprocal herkogamy or occurrence of H-morph) was unrelated to physiological self-incompatibility system breakdown. This phenomenon that floral morphological variation was independent of physiological self-incompatibility system provides an example for further studying the correlation between heterostylous morphology and physiology.

## Data Availability

The original contributions presented in the study are included in the article/**Supplementary Material**. Further inquiries can be directed to the corresponding author.
