# Supplementary material for: Does the occurence of homostyly necessarily accompany the breakdown of heteromorphic incompatibility system?
Source: Front Plant Sci. 2025 Feb 27;16:1402333. doi: 10.3389/fpls.2025.1402333 (PMC11903752; doi:10.3389/fpls.2025.1402333)
Supplement: Supplementary file 1 [file Table1.docx]

***Supplementary Material***

**Jing Zhao,** **Laititi Kuliku, Aiqin Zhang*,FangFang Jiao, Dengfu Ren**

***Correspondence:** Aiqin Zhang,Email: [zhangaq@xju.edu.cn](mailto:zhangaq@xju.edu.cn)

**Supplementary Tables**

**Table S1** The floral morphs and floral morph frequency in different distributions of *L.aureum*

| Populations | Locations and altitudes | Cob. | | | Floral morph  frequency | Pap. | | | Floral morph  frequency | The proportation of  Cob / Pap. |
| --- | --- | --- | --- | --- | --- | --- | --- | --- | --- | --- |
|  |  | L | AH | H_L_ | (L: AH: H_L_) | S | RH | H_S_ | (S: RH: H_S_) | Morphs |
| WLKSY | N36°18′15.7″ E80°48′3.64″H=2346 m | 138 | 49 | 27 | 5.11:1.81:1 | 119 | 102 | 45 | 2.64:2.27:1 | 1:1.24(G=5.644 ***P*=0.018**) |
| NE | N36°21′48.4″E81°3′7.8″ H=2065 m | 101 | 30 | 37 | 3.37:1:1.23 | 70 | 41 | 46 | 1.71:1:1.12 | 1:0.93(G=0.372 *P*=0.542) |
| MF | N36°48′23.5″E83°0′14.0″H=1993 m | 69 | 15 | 35 | 4.6:1:2.33 | 51 | 23 | 37 | 2.22:1:1.61 | 1:0.93(G=0.278 *P*=0.598) |
| QM | N37°39′16.1″ E86°6′42.2″H=2480 m | 83 | 11 | 5 | 16.6:2.2:1 | 27 | 35 | 21 | 1.29:1.67:1 | 1:0.84(G=1.408 *P*=0.235) |
| ATS | N76°64′36.9″E39°77′9.4″H=1207 m | 0 | 5 | 43 | 0:1:8.6 | 0 | 13 | 41 | 0:1:3.15 | 1:1.13(G=3.353 *P*=0.552) |
